# Why Can’t I Resist Those “Puppy Dog” (or “Kitty Cat”) Eyes? A Study of Owner Attachment and Factors Associated with Pet Obesity

**DOI:** 10.3390/ani11020539

**Published:** 2021-02-19

**Authors:** Anthony E. Coy, Jeffrey D Green, Anna Maria C. Behler

**Affiliations:** 1 Department of Psychology, University of South Florida, Tampa, FL 33620, USA; 2 Department of Psychology, Virginia Commonwealth University, Richmond, VA 23284, USA; jdgreen@vcu.edu; 3 Department of Psychology, North Carolina State University, Raleigh, NC 27607, USA; acbehler@ncsu.edu

**Keywords:** human-animal interactions, attachment, anxiety, pet weight, pet obesity

## Abstract

**Simple Summary:**

Attachment theory has become widely used in research on human-animal interactions. However, the majority of this research has examined how individual differences in attachment affect how humans seek care and comfort from animals. The current paper examines an additional component of attachment theory—the caregiving component. Specifically, the aim of the study was to understand how individual differences in attachment anxiety and avoidance predict factors associated with pet obesity including pet weight, body condition, daily treats, and daily interactions. An online survey with recruitment via social media captured the responses of dog and cat owners. As expected, all of the associations between owner attachment and factors associated with pet obesity were mediated by the owner’s concerns that a pet may negatively evaluate them and the owner self-reported caregiving and attentiveness. That said, the results indicate that higher levels of attachment anxiety were associated with dog and cat owners providing a greater number of daily treats and more daily interaction, and a larger body condition for dogs. In addition, higher levels of attachment avoidance were associated with dog (but not cat) owners giving fewer treats and less daily interaction. Interestingly, despite these relationships, pet weight was not associated with owner attachment. Although the effects presented in the present paper are relatively small, they are nonetheless important, as continuing to understand how differences in attachment anxiety and avoidance are related to care-giving behavior and pet obesity may provide for novel interventions that veterinarians can use to improve pet well-being and may provide avenues for future research.

**Abstract:**

Attachment theory posits that patterns of interaction derived from the attachment system provide a starting point for understanding how people both receive and provide care. Extending this theory to human-animal interactions provides insights into how human psychology affects pets, such as pet obesity. The goal of this study was to determine how attachment anxiety and avoidance might contribute to pet obesity. We assessed 563 pet owners’ attachment-related anxiety and avoidance, as well as additional attachment-related constructs (emotional rejection, evaluation concern, caregiving, and attentiveness to a pet). We also assessed various factors associated with pet obesity, including weight, body condition, daily treats, and daily interaction. The results indicate that dog owners high in attachment anxiety are concerned about how their pet may evaluate them, leading to more caregiving and attentiveness that results in more treats given per day, and a larger body condition (but not weight). In addition, owners high in attachment avoidance may seek to downplay the possibility of the dog negatively evaluating them, thus providing more negligent care. These findings suggest that attachment plays a unique role in shaping the pet-caregiver relationship and influences various elements that contribute to pet obesity, particularly in dogs. As such, the findings may lend a novel perspective to strategies for reducing pet obesity and provide a framework for future research into pet health.

## 1. Introduction

Recent research suggests that aspects of human psychology may drive how people care for animals [1,2]. Specifically, attachment theory has been used to examine caregiving and attentiveness towards pets [1]. However, the research on this topic has generally only examined self-reported owner behaviors (for example, attentiveness), rather than more pet-relevant outcomes. The present research is specifically interested in how owner attachment may affect pet obesity, which continues to be ever more prevalent, especially in dogs [3]. This can have detrimental effects on pet health including diabetes, cardiovascular disorder [4], and shorter life expectancy [5]. Although recent research has begun to recognize the extent to which humans contribute to pet obesity, most interventions tend to focus on exercise and diet of the pet [6,7,8]. This research seeks to provide an initial understanding of how owner attachment affects pet obesity, which may provide a novel perspective on the underlying factors owners contribute to the well-being of their pets. 

### 1.1. Attachment Theory

Attachment theory specifies that the way in which people seek care from others is developed from the nature of emotional bonds with their earliest caregivers [9,10]. These initial patterns provide fodder for mental models of the relationship between the individual and the outside world. These attachment orientations persist throughout a person’s life, and ultimately inform how a person relates to and provides care for others. Attachment behaviors are based upon two dimensions of attachment: anxiety and avoidance [11,12]. The anxiety dimension indicates a person’s level of concern with being accepted or rejected by others. At high levels, anxious attachment can manifest itself via the individual feeling great concern that a close other may view them negatively and want to end a relationship. This can result in anxious individuals paying increased attention towards others in an attempt to reduce odds of rejection [11,12]. The avoidance element of attachment is related to a person’s level of comfort with intimacy and dependence, with more avoidant individuals feeling significant discomfort with emotional closeness. They are also less likely to give support to others, and have a tendency to see support-seeking as weakness [12].

In addition to individual differences in attachment anxiety and avoidance, attachment theory specifies three fundamental attachment systems—careseeking, exploration, and caregiving—through which differences in behavior may be observed [11,12]. The careseeking system seems to receive the vast majority of attention, especially in research on human-animal interactions. This system articulates how people turn to attachment partners (e.g., children to parents, adults to romantic partners) for comfort in times of distress, depending on levels of avoidance and anxiety [11,12]. More specifically, highly anxious people tend to become distressed easily and seek comfort to validate their worth, whereas highly avoidant individuals turn inward because prior experiences have shaped their view that others cannot be trusted. The exploration system has received far less attention but specifies that people use attachment partners as a secure base from which to explore their world [11,12,13]. 

Most pertinent to the present research is the caregiving system. This system drives how individuals provide care for attachment partners, particularly in times when the partner is distressed. Although secure individuals are able to accurately identify distress and provide the support needed, highly anxious and avoidant individuals have greater difficulty with caregiving [12]. Specifically, highly anxious individuals attempt to prove their worth when providing care [14], resulting in more aversive and less effective caregiving because their responses are more self-focused [15,16,17,18]. Highly avoidant individuals are generally poor caregivers [18,19] because they are lower in prosocial motivation and lower in emotional intimacy with their partner [12], which is necessary for more targeted and appropriate support-giving. We sought to determine whether pet well-being is affected by attachment-based caregiving behaviors and, if so, the extent to which attachment anxiety and avoidance influence these behaviors.

### 1.2. Attachment and Human-Animal Interactions

Pets are cherished members of millions of households. The American Pet Products Association [20] reports that approximately 67% of US households have a pet. Many owners consider their pet to be a friend or family member [21,22], both of which are attachment-relevant relationships. In fact, people draw upon relationships with pets as a source of self-esteem and self-worth [23], and the emotional bonds formed with pets [24,25] can become strong enough to result in feelings of extreme grief upon their passing, similar to experiencing the loss of a human loved one [26]. Given that pet owners make up such a sizable part of the population, and that pet ownership is such a unique experience that combines aspects of various types of close relationships, a better understanding of how human psychology—and specifically attachment—affects pets is crucial to ensuring the well-being of these animals. 

Previous research found evidence that aspects of attachment apply to human-animal interactions. Specifically, the careseeking system has been particularly well studied to identify the benefits pets may provide in terms of stress and anxiety reduction, including short-term reductions in stress associated with finals in college students [27] and longer-term reductions in stress over 2.5 years, associated with dog ownership for parents of children with autism spectrum disorder [28]. Further, teenagers with stronger pet attachment used more adaptive coping strategies when under a chronic stressor like parental military deployment [29]. 

Although these effects clearly demonstrate the tremendous importance animals have in the lives of humans, less work on attachment has examined the influence humans have on their animals. Previous studies have explored how people make decisions regarding care in emergencies [30] and factors that predict animal cruelty [31]. We know of only one study that explicitly focused on attachment and pet caregiving [1], finding that both attachment anxiety and avoidance influenced self-reported caregiving and attentiveness, with highly anxious individuals reporting greater attentiveness and caregiving because they were concerned about their pet evaluating them negatively. In contrast, highly avoidant individuals reported less caregiving and careseeking behaviors because of this concern. The present research seeks to expand this model to include pet well-being outcomes. As previously mentioned, pet obesity is one condition that influences pet welfare [4,5] and has become increasingly common [3]. As such we sought to assess pet weight and body condition along with associated environmental factors that contribute to pet obesity, including daily interaction with one’s animal and the number of treats given each day [32,33]. Previous research found that owner reports of exercise were associated with a lesser likelihood of obesity in dogs, and obese dogs were given treats more often than normal weight dogs [33]. In this study, we expand exercise to “interactions” to allow for greater reporting of daily interactions, including from cat owners who may not feel that exercise is an applicable description to the interactions they have with their animals. Importantly, this may expand on the types of interactions that are reported for dogs as well, beyond exercise. This change in conceptualization (that is, exercise vs. interaction) may shift the relationship between daily interactions, as measured in the present study, and the other factors associated with pet obesity. Additionally, the current conceptualization may be particularly relevant to attachment. That is, as previous research has demonstrated that highly anxious individuals tend to provide care to demonstrate their own self-worth and in an intrusive fashion, it is possible that highly anxious individuals will seek out opportunities to comfort their pet—thus showing greater interaction, but not necessarily exercise. 

### 1.3. Current Research

**Figure 1:**
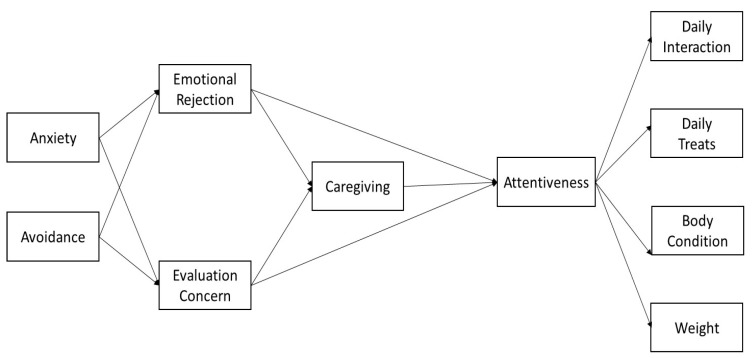
Conceptual model.

Given the seemingly universal nature of attachment, and that previous research has demonstrated that attachment dynamics apply to human-animal relationships, it is logical to expect that caregiving towards pets would be influenced by attachment anxiety and avoidance. Thus, the current work focused on two specific research questions. First, we sought to explore how attachment-related concepts (for example, rejection-sensitivity, caregiving, and attentiveness) were related to factors associated with pet obesity. Second, we were interested in how pet well-being was tied to attachment anxiety and avoidance. Exploring these questions would allow us to learn more about the ways that human attachment can influence health and wellness outcomes for pets. Given that dogs and cats are the most common types of household pets, and that we tend to form more social relationships with them relative to other types of animals, we specifically sampled owners who reported owning either of these types of pets. We sought to test these questions by extending a framework of pet-focused caregiving behaviors [1] to pet well-being outcomes, including daily treats given, daily interaction, pet weight and body condition (see Figure 1 for the conceptual model). We had two specific hypotheses for our study:

**Hypothesis** **1.** *Owners with high attachment anxiety will report higher levels of factors related to pet obesity (i.e., body condition, weight, daily treats given, and daily interaction), relative to owners with low attachment anxiety, as mediated by owner rejection sensitivity, caregiving, and attentiveness*.

**Hypothesis** **2.** *Owners with high attachment avoidance will report lower levels of factors related to pet obesity (i.e., body condition, weight, daily treats given, and daily interaction), relative to those with low attachment avoidance, as mediated by owner rejection sensitivity, caregiving, and attentiveness*.

## 2. Methods

The local institutional review board reviewed and approved this work. Prior to participating in the online study, participants reviewed the consent form displayed on the first page and provided consent by continuing with the survey. Participants were not compensated for completing the study.

### 2.1. Participants

Participants were recruited via social media when researchers posted recruitment information on the “wall” of animal interest groups (with permission as applicable) and via online distribution by associates of the researchers. Participants were required to be at least 18 years of age and had to be the current primary caretaker of a pet for at least 2 months in order to participate.

### 2.2. Procedure and Measures

Participants first completed the surveys on attachment anxiety and avoidance, then pet-related attachment constructs, and finally answered questions related to the well-being of their pet. Participants were specifically instructed to respond to the items with the pet they felt most attached to. After completing the survey, participants read an online debriefing describing the purpose of the study. Descriptive statistics and Cronbach’s alpha coefficients for all measures are reported in Table 1, along with correlations between all measures.

### 2.3. Attachment

The general version of the Experiences in Close Relationships-Revised scale (ECR-R, [34]) was used to assess attachment-related anxiety (for example, “I’m afraid that I will lose my partner’s love”) and avoidance (for example, “I prefer not to show a partner how I feel deep down”). Items were measured on a 7-point numeric rating scale with the endpoints 1 (Strongly Disagree) and 7 (Strongly Agree). The use of this measure replicates previous research [1,35] and was used to most effectively evaluate how global attachment dispositions are linked to pet-related caregiving behaviors and pet health.

### 2.4. Attachment Related Constructs

Four scales were used to assess pet-specified, attachment-related constructs. Specifically, the emotional rejection (for example, “I never worry about being rejected by my pet”) and evaluation concern (for example, “I don’t want my pet to think badly of me”). Subscales of the pet-specific rejection sensitivity measure [1] each contained three-items that assessed the thoughts and concerns people had regarding their pets. Caregiving was measured using a three-item scale designed to assess the degree to which an owner attends to a pet’s emotional needs (for example, “When my pet is upset, I do my best to calm my pet down”; [35]). Finally, pet-specific attentiveness was assessed using four items that sought to evaluate the extent to which individuals attended to the physical needs of their pet (for example, “I frequently buy new things for my pet”). Each of the four surveys was measured on a 7-point numeric rating scale with the endpoints 1 (Strongly Disagree) and 7 (Strongly Agree).

### 2.5. Pet Specific Measures

Participants also reported the breed, sex, and medical conditions of their pet, along with a series of single-item measures that were used as outcome measures related to pet health and well-being. Specifically, pet weight, body condition, the typical number of treats given per day (i.e., daily treats), and the typical number of hours they spent interacting with their pet each day (that is, daily interaction). For pet weight, we converted the weight provided to z-scores using the average weight and range provided in Bell and colleagues [36] Veterinary Medical Guide to Dog and Cat Breeds. As cat owners were less clear on the specific breed of cat in their reports relative to dog owners, pet weights were only evaluated for dogs. In cases where only the range for the breed was reported, the mid-point of the range was used as the average. It was assumed the range represented one standard deviation around the mean when calculating z-scores for weight. When a breed’s weight was not reported, an internet search was conducted to identify the necessary information and, failing that, we excluded the pet from that analysis (*n* = 68 dogs). For mixed breeds, we assumed the breed noted (or noted first) was the dominant breed (*n* = 122 dogs). We note that these two factors are not mutually exclusive as some participants merely reported “mixed” dogs. However, the results for standardized pet weight are consistent regardless of whether mixed breeds were included in the analysis and excluding them would severely limit the sample size (see results for pet-specific information). Additionally, we included a measure of body condition that could be used for all dogs and cats, regardless of breed. Specifically, the measure of body condition was a 5-point visual scale and participants reported which image of a pet best indicated their animal’s body condition, with images included for dogs and cats. On this scale, lower numbers represent a thinner pet, and higher numbers represent a more overweight pet, with the midpoint being an ideally healthy body condition. For daily number of treats and daily time spent interacting, when a participant reported a range rather than a specific number, the low end of the range was used as the number as some participants only reported a low end of a range (e.g., 8+ treats) for these variables rather than a specific number or true range. 

## 3. Results

### 3.1. Participant Demographics

There were 563 individuals who completed the online survey; 85% (*n* = 477) were female, 11% (*N* = 60) were male and 26 participants did not report their sex. The age of the sample individuals averaged 38.80 years old (*SD* = 13.49, range 18–78 years), 87% (*n* = 488) self-reported as Caucasian, 3% (*n* = 18) self-reported as Asian American, 3% (*n* = 15) self-reported as Hispanic/Latino, 1% (*n* = 8) self-reported as Black or African American, 6% (*n* = 34) self-reported as another race or chose not to self-identify.

### 3.2. Pet Information

The majority of participants 68% (*n* = 384) reported pet-related information about a dog, 25% (*n* = 138) reported about a cat, and 7% (*n* = 41) reported on another type of animal. Most (69%, *n* = 387) indicated a single breed for the animal, though 150 (27%) participants reported information on a mixed breed animal, including a miniature breed, with the remaining participants reporting an animal other than a dog or a cat, or reporting on more than one animal. Participants reporting on an animal other than a dog or cat, or reporting on more than one animal, were removed from the analyses as were participants who reported their pet as having a medical condition that influenced weight (for example, heartworm survivor, digestive issues). This left 493 participants in the dataset for the analyses. 

### 3.3. Statistical Analyses

Prior to conducting the planned path analyses we first examined the basic correlations presented in Table 1. The majority of these are consistent with our expectations based on previous research. However, we note that standardized weight was not significantly related to any variable other than body condition (r = 0.13). Similarly, body condition was only related to weight and evaluation concern (r = 0.12). However, we note that research has shown mediation analyses do not require direct effects [37,38] and therefore proceeded with the analyses. 

Using path analysis, we tested our hypotheses that higher levels of attachment anxiety (H1) and avoidance (H2) would be related to poorer pet well-being (i.e., body condition, weight, treats given per day), mediated by pet-related rejection sensitivity and attachment-related caregiving and attentiveness. Models were run separately for dogs and cats as cat owners were less clear on the specific breed of cat in their reports relative to dog owners. This lack of clarity made it difficult to standardize weights for cats and we elected to omit weight from the analysis for cats.

For dogs, the initial model was a good fit to the data, *χ*^2^(25) = 34.55, *p* = 0.09; comparative fit index (CFI) = 0.96; Tucker–Lewis index (TLI) = 0.93; root mean square error of approximation (RMSEA) = 0.04, 90% CI [.00, 0.07]; and standardized root mean square residual (SRMR) = 0.05 with the results for direct effects in Table 2 and indirect paths in Table 3. Neither attachment anxiety nor avoidance predicted emotional rejection but both anxiety and avoidance predicted evaluation concern. In turn, evaluation concern predicted caregiving behavior and both caregiving behavior and evaluation concern predicted attentiveness. Greater attentiveness then predicted greater number of treats given (*R*^2^ = 0.17), and a greater daily interaction (*R*^2^ = 0.09) with the dog. Additionally, the relationship between attentiveness and body condition was trending (*p* = 0.06; *R*^2^ = 0.01) in the expected direction. However, this was not the case with breed-standardized pet weight. It is noteworthy that when we exclude breed−standardized weight from the model, the relationship between attentiveness and body condition was significant (*p* = 0.04). This is likely due to the number of participants that were removed from the analysis that included weight, as we were unable to find the average weight and range for some dog breeds. Importantly, the size of the effect changes only slightly without standardized weight (0.067 vs. 0.069), indicating stability in the estimate and significance changing as a function of the number of participants. Indirect effects indicated that attachment anxiety and avoidance both influenced daily treats given and daily interaction with dogs, albeit in opposite directions. As expected, high attachment anxiety ultimately predicted more daily treats and daily interaction, whereas high attachment avoidance resulted in fewer treats and less daily interaction—both confirming our hypotheses regarding dogs. For cats, this model was a relatively poor fit to the data, *χ*^2^(20) = 39.28, *p* = *0*.006; (CFI) = 0.87; TLI = 0.76; RMSEA = 0.09, 90% CI [0.05, 0.14]; and SRMR = 0.07 with the results for direct effects in Table 2 and indirect paths in Table 3. Although a relatively poor fit to the data.

We note that the effect for attachment anxiety ultimately predicted more daily treats given (*R*^2^ = 0.19) and daily interaction with the cat (*R*^2^ = 0.05). Despite attempts to modify the model for cats, none proved effective at increasing the fit indices. Thus, when it comes to cats the data provided some tentative support for our hypothesis on attachment anxiety (H1), but not avoidance (H2), but the poor model fit makes it difficult to draw firm conclusions. However, we note that these effects remain significant in a model with cats and dogs combined, and with only body condition, daily treats, and daily interactions as outcomes. However, due to pet weight only being useful for dogs, we reported separate analyses.

## 4. Discussion

Much of the existing research on attachment theory and human-animal interactions has focused on the benefits humans receive from animals (that is, careseeking component of attachment; [39,40] and how these benefits vary based on dimensions of attachment [1,35]. Although prominent in research on attachment between humans, far less work has focused on the caregiving system of attachment theory when it comes to human-animal interactions. This study sought to elucidate this work by extending a previously established framework of caregiving [1] to animal-focused outcomes of pet weight, body condition, daily treats given, and daily interaction, each of which may be a component of pet obesity. The findings suggest that attachment anxiety predicts some of the pet obesity outcomes, including daily interaction and treats given, though we were only able to draw strong conclusions on these points for dogs. It may be argued that the effects for daily treats and daily interaction are inconsistent, as more treats given would lead to obesity whereas more interaction would lead to less. We note that previous research finding these effects examined daily exercise rather than all interactions [32]. This was used, in part, to allow cat owners to report interaction as “exercise”, which may be less common. The more general measure used in the present study likely allowed for more participants to report interactions who may not normally exercise their pet. Future research should seek to clarify if differences in these measures exist. Additionally, given the nature of anxious attachment as self-focused with the goal to prove ones worth, it is possible that the interaction assessed in the present research is actually excessive and may stress, rather than comfort, the animal. This interpretation is supported by the fact that the relationship between attachment anxiety and interactions is mediated by evaluation concern: the owner worrying that their pet may negatively evaluate them. Additionally, there is a trending association with a dog’s body condition wherein more anxious individuals report a dog with a larger body. Taken together, these findings suggest that anxious individuals may provide inappropriate and self-focused care for their pets, especially dogs.

Such findings are consistent with other research on attachment anxiety and interpersonal relationships. Specifically, highly anxious individuals tend to be overly concerned with the standard of care they provide in romantic relationships [15,41] and to children [42], and this preoccupation ultimately results in lower quality caregiving. Thus, it is logical that the same underlying process would yield similar results in human-animal interactions. Likewise, highly avoidant individuals tend to be negligent when it comes to caregiving and results for dogs appear consistent with this expectation. Moreover, these findings are consistent with previous research that only examined the human elements of the model. This consistency across studies further strengthens the argument that human attachment influences animal well-being.

It is noteworthy that the primary limitation of the study may have weakened the reported findings. Specifically, pet owners are untrained and therefore may be less accurate at reporting pet weight and body type [43,44]. Additionally, despite the anonymous nature of the survey, owners may be self-conscious about indicating their pet is overweight and possibly factors associated with their pet being overweight (for example, daily treats, daily interaction). These factors could potentially explain the lack of a significant relationship between daily treats given, interaction, and pet weight or body condition, as these variables would seem to be connected. That said, the number of daily treats could be associated with dog training or other non-weight associated factors. That is, highly anxious individuals concerned about caring for their pet may seek to have a well-trained pet and therefore report providing a greater number of treats each day. Likewise, training may also be associated with greater time associated with daily interactions. Thus, a collaborative effort between psychologists and veterinarians wherein the veterinarians providing indicators of pet well-being—including medical and training aspects in addition to weight and body condition—may offer a more complete picture of how human attachment affects pet well-being. This would also eliminate any potential recall bias that may exist for all of the outcomes in the study. Additionally, veterinarians may be better able to identify cat breeds and weights to allow for a stronger test of the model with cat owners. 

Another limitation was the focus on the single pet the individual felt most attached to, rather than all of the animals an individual may have. It may be beneficial to determine if the effects hold across all pets. Indeed, recent research demonstrated that having a non-preferred pet in the home may lower well-being for humans [45], and it may make sense that caregiving towards such pets may look different than it does towards the animal to which the person feels most attached. Lastly, the cross-sectional and correlational nature of the data is an additional limitation of this design. A longitudinal study that tracked pet well-being may be useful, especially if it tracked the pet from initial adoption by an owner to see changes over time as a result of ownership and specifically owner attachment anxiety and avoidance.

The primary implication of the present research is that factors related to healthy body weight, are driven, in part, by the attachment dynamics of their owners. Specifically, owners high in attachment anxiety have greater concern about their pet’s evaluation of them which, in turn, drives attentiveness and caregiving behaviors. For veterinarians, this suggests that dietary education alone may not be enough to change behavior in owners with overweight pets. That is, owners may understand the dietary concerns but still feel an emotional pull to maintain a connection with their pet, ultimately leading to the owner disregarding the recommendations. Although the effects are relatively small in the context of the current research and survey, it is possible such feelings of concern may have cumulative effects throughout and perhaps even across days (that is, a pet “begs” for a treat multiple times and eventually gets one). Further, the findings offer a novel factor that contribute to pet-directed behaviors, suggesting that reassuring highly anxious owners that a pet will not negatively evaluate them for following certain restrictions may create greater adherence to these recommendations. Such interventions could be further developed by connecting them with attachment-based interventions that have shown promise in interpersonal/romantic relationships. For example, the Attachment Security Enhancement Model (ASEM) by Arriaga and colleagues [46] highlights building personal confidence and building more secure mental models of the self, particularly for those who are highly anxious. Researchers could experimentally test such interventions to determine if they are more effective than existing approaches. Similar interventions may be implemented for other instances in which excessive “care” of the animal is a concern (for example, overstimulation). 

## 5. Conclusions

In summary, the present study replicated and extended an attachment-focused model of caregiving for pets. The findings suggest that individuals high in attachment anxiety have concerns that their pet may negatively evaluate them. In turn, this concern drives caregiving and attentiveness behaviors that ultimately predict elements of pet well-being, specifically daily treats given, daily time interacting, and body condition for dogs. In contrast, individuals high in attachment avoidance may seek to downplay these feelings and provide negligent care for their animal (specifically their dog). Although the effects presented in the present paper are relatively small, they are nonetheless important, as continuing to understand how differences in attachment anxiety and avoidance are related to caregiving behavior and pet well-being may provide for novel interventions that veterinarians can use to improve pet well-being. Overall, the findings make it clear that human attachment affects pet well-being and future research is needed to further clarify these effects.

## Figures and Tables

**Table 1:** Means, standard deviations, and intercorrelations among measures.

Scale	1	2	3	4	5	6	7	8	9	10
*M* =	2.81	2.74	6.37	4.12	6.27	4.96	4.53	10.00	0.86	2.99
*SD* =	1.47	1.27	0.92	1.57	0.87	1.50	3.60	6.43	4.59	0.96
α =	0.92	0.90	0.67	0.63	0.69	0.80	─	─	─	─
1. Anxiety	─									
2. Avoidance	0.41***	─								
3. Emotional Rejection	−0.003	−0.11 *	─							
4. Evaluation Concern	0.27 ***	0.04	0.06	─						
5. Caregiving	0.04	−0.07	0.37 ***	0.32 ***	─					
6. Attentiveness	0.13 *	−0.03	0.27 ***	0.30 ***	0.53 ***	─				
7. Daily Interaction	0.05	0.12 *	0.15 ***	0.12**	0.26 ***	0.30 ***	─			
8. Daily Treats	−0.01	−0.05	0.09	0.06	0.15 **	0.44 ***	0.23 ***	─		
9. Weight (Standardized)	−0.07	−0.04	−0.02	0.03	0.01	0.04	−0.08	0.06	─	
10. Body Condition	0.05	−0.02	0.03	0.12 **	0.09	0.07	0.003	−0.02	0.13 *	─

Note. *** *p* < 0.001; ** *p* < 0.01; * *p* < 0.05.

**Table 2:** Path statistics for dog and cat owner models.

			Dogs					Cats		
Direct Effects (Outcome) (Predictor)	Est.	SE	*p*	CI LL	CI UL	Est.	SE	*p*	CI LL	CI UL
Weight (Breed Standardized)										
Attentiveness	0.10	0.19	0.60	−0.28	0.48	-	-	-	-	-
Daily Treats										
Attentiveness	1.74	0.23	0.00	1.28	2.20	1.67	0.34	0.00	1.01	2.34
Daily Interaction										
Attentiveness	0.76	0.14	0.00	0.48	1.04	0.42	0.17	0.01	0.08	0.76
Body Condition										
Attentiveness	0.07	0.04	0.06	0.00	0.14	0.08	0.07	0.22	−0.05	0.22
Attentiveness										
Caregiving	0.76	0.10	0.00	0.57	0.95	0.66	0.16	0.00	0.35	0.98
Evaluation Concern	0.15	0.05	0.00	0.05	0.25	0.19	0.09	0.03	0.02	0.37
Emotional Rejection	0.14	0.11	0.21	−0.08	0.36	0.17	0.11	0.12	−0.04	0.38
Caregiving										
Evaluation Concern	0.17	0.03	0.00	0.11	0.23	0.24	0.05	0.00	0.14	0.33
Emotional Rejection	0.26	0.07	0.00	0.12	0.39	0.24	0.06	0.00	0.12	0.36
Emotional Rejection										
Anxiety	0.03	0.03	0.40	−0.04	0.09	0.16	0.08	0.05	0.00	0.31
Avoidance	−0.05	0.04	0.19	−0.12	0.02	−0.24	0.10	0.02	−0.44	−0.04
Evaluation Concern										
Anxiety	0.31	0.07	0.00	0.17	0.46	0.43	0.09	0.00	0.26	0.61
Avoidance	−0.19	0.08	0.02	−0.34	−0.03	0.10	0.12	0.42	−0.14	0.33
Covariance	Est.	SE	*p*	CI LL	CI UL	Est.	SE	*p*	CI LL	CI UL
Weight (Breed Standardized)										
Daily Treats	0.95	1.63	0.56	−2.24	4.13	-	-	-	-	-
Daily Interaction	−1.71	1.01	0.09	−3.68	0.26	-	-	-	-	-
Body Condition	0.42	0.25	0.09	−0.07	0.90	-	-	-	-	-
Daily Treats										
Daily Interaction	1.13	1.20	0.35	−1.23	3.49	1.68	1.28	0.19	−0.84	4.19
Body Condition	0.01	0.30	0.99	−0.58	0.59	−0.21	0.51	0.68	−1.21	0.79
Daily Interaction										
Body Condition	0.03	0.18	0.89	−0.33	0.38	0.01	0.26	0.96	−0.49	0.52

**Table 3:** Indirect effects of attachment on attentiveness.

	Dogs					Cats				
Indirect Effects	Est.	SE	*p*	CI LL	CI UL	Est.	SE	*p*	CI LL	CI UL
Anxiety -> Evaluation Concern -> Caregiving	0.05	0.02	0.00	0.02	0.08	0.10	0.03	0.00	0.05	0.15
Anxiety -> Evaluation Concern -> Caregiving -> Attentiveness	0.04	0.01	0.00	0.02	0.06	0.06	0.02	0.00	0.02	0.10
Anxiety -> Evaluation Concern -> Caregiving -> Attentiveness -> Body Condition	0.00	0.00	0.10	0.00	0.01	0.01	0.01	0.26	0.00	0.01
Anxiety -> Evaluation Concern -> Caregiving -> Attentiveness - Daily Interaction	0.03	0.01	0.00	0.01	0.05	0.03	0.01	0.06	0.00	0.05
Anxiety -> Evaluation Concern -> Caregiving -> Attentiveness -> Daily Treats	0.07	0.02	0.00	0.03	0.11	0.11	0.04	0.01	0.02	0.19
Avoidance -> Evaluation Concern -> Caregiving -> Attentiveness -> Body Condition	0.00	0.00	0.16	0.00	0.00	0.00	0.00	0.51	0.00	0.01
Avoidance -> Evaluation Concern -> Caregiving -> Attentiveness - Daily Interaction	−0.02	0.01	0.05	−0.04	0.00	0.01	0.01	0.46	−0.01	0.02
Avoidance -> Evaluation Concern -> Caregiving -> Attentiveness -> Daily Treats	−0.04	0.02	0.04	−0.08	0.00	0.02	0.03	0.44	−0.04	0.08

## Data Availability

The data used in the study is available upon request by emailing the corresponding author.
